# miR-193a-3p increases glycolysis under hypoxia by facilitating Akt phosphorylation and PFKFB3 activation in human macrophages

**DOI:** 10.1007/s00018-022-04146-z

**Published:** 2022-01-24

**Authors:** Dominik C. Fuhrmann, Bernhard Brüne

**Affiliations:** 1 Institute of Biochemistry I, Faculty of Medicine, Goethe-University Frankfurt, Theodor-Stern-Kai 7, 60590 Frankfurt, Germany; 2 Frankfurt Cancer Institute, Goethe-University Frankfurt, Theodor-Stern-Kai 7, 60590 Frankfurt, Germany; 3 German Cancer Consortium (DKTK), Partner Site, Frankfurt, Germany; 4 Fraunhofer Institute for Translational Medicine and Pharmacology ITMP, Frankfurt, Germany

**Keywords:** PPTC7, mTOR, PDPK1, HIF

## Abstract

Human macrophages infiltrating hypoxic regions alter their metabolism, because oxygen becomes limited. Increased glycolysis is one of the most common cellular adaptations to hypoxia and mostly is regulated via hypoxia-inducible factor (HIF) and RAC-alpha serine/threonine–protein kinase (Akt) signaling, which gets activated under reduced oxygen content. We noticed that micro RNA (miR)-193a-3p enhances Akt phosphorylation at threonine 308 under hypoxia. In detail, miR-193a-3p suppresses the protein abundance of phosphatase PTC7 homolog (PPTC7), which in turn increases Akt phosphorylation. Lowering PPTC7 expression by siRNA or overexpressing miR-193a-3p increases Akt phosphorylation. Vice versa, inhibition of miR-193a-3p attenuates Akt activation and prevents a subsequent increase of glycolysis under hypoxia. Excluding effects of miR-193a-3p and Akt on HIF expression, stabilization, and function, we noticed phosphorylation of 6 phosphofructo-2-kinase/fructose 2,6-bisphosphatase PFKFB3 in response to the PI3K/Akt/mTOR signaling cascade. Inhibition of PFKFB3 blocked an increased glycolytic flux under hypoxia. Apparently, miR-193a-3p balances Akt phosphorylation and dephosphorylation by affecting PPTC7 protein amount. Suppression of PPTC7 increases Akt activation and phosphorylation of PFKFB3, which culminates in higher rates of glycolysis under hypoxia.

**Supplementary Information:**

The online version contains supplementary material available at 10.1007/s00018-022-04146-z.

## Introduction

Hypoxia occurs when the demand of oxygen exceeds its supply. This happens for example in tumors, diabetes, or inflammation. Adaptation to hypoxia is a process, which provokes alterations in metabolism. We can discriminate between different degrees of hypoxia, i.e., acute versus chronic hypoxia [1]. These stages of hypoxia are characterized by distinct metabolic adaptations. Under acute hypoxia, glycolysis increases and pyruvate is used as the major metabolite for oxidative phosphorylation. The activity of the respiratory chain decreases under hypoxia by inactivation of complex I and an altered composition of complex IV. However, mitochondrial membrane potential and thus integrity is preserved [2–4]. During chronic hypoxic cells relay on glutamine and fatty acids to maintain electron transport. To coordinate these adaptive responses, different regulatory mechanisms are in place. Under acute hypoxia, hypoxia-inducible factor (HIF)-1α is a major transcriptional regulator to adjust metabolism and responsible for modifications in respiratory chain complexes and increased glycolysis [5]. Under chronic hypoxia HIF-2α appears to be more prominent [6]. HIF-1 activates transcription of genes coding for glycolytic enzymes, such as phosphofructokinase (PFK), aldolase, phosphoglycerate kinase-1, enolase, lactate dehydrogenase A (LDHA), and pyruvate dehydrogenase kinase 1 (PDK1), which inactivates pyruvate dehydrogenase (PDH) and limits citric acid cycle activity [7]. Also, 6 phosphofructo-2-kinase/fructose 2,6-bisphosphatase (PFKFB) 3 was shown to be HIF-1 responsive [8]. Phosphorylation and thus activity of PFKFB enzymes increased in response to the phosphatidylinositol 3-kinase (PI3K)/RAC-alpha serine/threonine–protein kinase (Akt) pathway under hypoxia and this contributes to hypoxia-induced glycolysis [9–11]. Four PFKFB isoforms catalyze the synthesis and degradation of fructose 2,6-bisphosphate from or to fructose 6-phosphate [12]. Fructose 2,6-bisphosphate in turn enhances the catalytic activity of PFKs and consequently the production of fructose 1,6-bisphosphate, which comprises the rate limiting step in glycolysis. In addition to its role in PFKFB activation, Akt increases the expression of glucose transporter (Glut) 1, which facilitates glucose uptake [13] and promotes hexokinase (HK) translocation to the mitochondrial membrane, in association with its increased activity [14].

Akt can directly be phosphorylated at serine and threonine residues by mammalian target of rapamycin complex (mTORC) 2 and 3-phosphoinositide-dependent protein kinase 1 (PDPK1). Of note, Akt activity can be reduced by phosphatidylinositol 3,4,5-trisphosphate 3-phosphatase and dual-specificity protein phosphatase (PTEN), which antagonizes PI3K activity. In addition, Akt activity is decreased by dephosphorylation by, e.g., serine/threonine–protein phosphatase 2A [15].

The metabolism of macrophages is easily adjusted by environmental cues and linked to macrophage polarization [16]. Macrophages facing hypoxia execute a wide spectrum of pro- and anti-inflammatory responses including tissue regeneration [17, 18]. Consequently, the metabolism of human macrophages needs to be adjust, which makes them an interesting cell to investigate adaptive responses to hypoxia. Unfortunately, underlying molecular mechanisms are not fully understood.

A further component of hypoxic adaptation are micro RNAs (miR). miR-210 is a well-studied hypoxia-inducible miR, which regulates the expression of various target gens, e.g., the iron–sulfur cluster assembly enzyme ISCU [19]. ISCU promote the incorporation of iron–sulfur clusters into apoproteins and has a pivotal role in metabolism [20]. While the role of miR-210 in metabolism under hypoxia is well established, data on the miR-193 family in this context are rare. In glioblastoma cells miR-193a-3p is HIF-1-dependently induced [21] and a protective function of miR-193a-3p inhibition during intermittent hypoxia was shown in endothelial cells [22]. In general, the role of the miR-193 family under hypoxia is largely unknown with the notion that primarily miR-193a-3p but not miR-193b-3p was correlated to hypoxia. The miR-193 family was recently described as a tumor suppressor, e.g., by targeting receptor tyrosine–protein kinase erbB-4, p21, or the Akt pathway [23–27]. During macrophage activation, miR-193a-3p was shown to regulate NF-κB signaling upon lipopolysaccharide treatment in lung fibroblasts by decreasing toll-like receptor 4 and consequently cytokine expression [28]. In addition, miR-193a-3p was shown to target PTEN and thus, has the ability to increase Akt activity [29, 30]. Taken together, a major focus of miR-193 referred to tumor progression, while its role in metabolism during hypoxia in macrophages is less clear. In our study, we identified miR-193a-3p to target protein phosphatase PTC7 homolog (PPTC7) and thus, Akt activation under hypoxia. In turn, Akt activated PFKFB3, which substantially adds to control glycolysis under hypoxia. We suggest that besides HIF-1α, the miR-193a-3p/PPTC7/Akt/PFKFB3 signaling cascade is crucial for increased glycolysis under hypoxia.

## Materials and methods

### Isolation of primary human monocytes

Primary human monocytes were isolated from Buffy coats from healthy donors using Leucosep tubes (Greiner bio-one, Frickenhausen, Germany) and Biocoll Separating Solution (Biochrom, Berlin, Germany). Cells were washed three times with PBS and were allowed to adhere to 6-well plates, 6 cm dishes, or 48-well plates (Cell + , Sarstedt, Nümbrecht, Germany) for 1 h at 37 °C. Non-adherent cells were removed and remaining monocytes were incubated for at least 7 days with RPMI 1640 medium containing 3% human serum and penicillin/ streptomycin for differentiation to macarophages. Macrophages were used at a density of approximately 80%. Since the Buffy coats were anonymized, no ethical statement was necessary.

### siRNA transfection

Primary human macrophages were transfected with 50 nM siRNA against PPTC7, HIF-1α, or HIF-2α (ON-TARGETplus SMART pool, human PPTC7, HIF-1α or HIF-2α), 25 nM miR-193a-3p antagomir (miRCURY, Qiagen, Hilden, Germany), or 10 nM miR-193a-3p mimic (Sigma-Aldrich, Munich, Germany) using HiPerFect transfection reagent (Qiagen, Hilden, Germany).

### Treatments

AKTVIII (1 µM), Torin-2 (100 µM), and LY294002 (15 µM) purchased from Sigma-Aldrich. GSK2334470 (5 µM) and 3-(3-Pyridinyl)-1-(4-pyridinyl)-2-propen-1-one (3PO, 10 µM) were purchased from Cayman Chemicals (Ann Arbor, USA). All inhibitors were added to the cells 1 h prior hypoxic incubation. Hypoxic incubation was performed in a SciTive Workstation (Baker Ruskinn, Leeds, UK) at 1% O_2_ and 5% CO_2_ for 16 h.

### Western analysis

Cells were lysed in a buffer containing 6.65 M urea, 10% glycerol, 1% SDS, 10 mM Tris/HCl, pH 7.4 and sonicated. Protein content was determined by a protein assay kit (Bio-Rad, Munich, Germany) and 60 µg protein was loaded on a 10% SDS gel. For HIF 7.5% SDS gels were used. Gels were blotted using a Trans Blot Turbo blotting system (Bio-Rad). Before blocking membranes were stained using the Revert™ 700 Total Protein Stain kit (Licor, Lincoln, USA) according to manufacturer’s instructions. Afterwards, membranes were blocked in 5% milk in TBS-T for Akt (9272, Cell Signaling, Frankfurt, Germany), pAkt Thr 308 (5106, Cell Signaling), pAkt Ser 473 (4051, Cell Signaling), PPTC7 (HPA039335, Sigma-Aldrich), HIF-1α (610959, BD, Heidelberg, Germany), PFKFB2 (39527, Cell Signaling), PFKFB2 Ser 483 (39527, Cell Signaling), PFKFB3 (ab181861, abcam, Cambridge, UK), and PFKFB3 Ser 461 (ab202291, abcam). Fluorescence signal was detected on an Odyssey scanner (Licor) and quantified with Image Studio Digits 5.0 (Licor). For each lane the lane normalization factor (LNF) was calculated (intensity of a complete lane divided by the intensity of the lane with the maximal intensity) and used for normalization of the signal of the corresponding primary antibody. Complete total protein stains are collectively shown in Figure S1.

### Real time PCR

RNA was isolated using Trizol (Thermo Fisher Scientific, Waltham, USA) and measured using a Nanodrop ND-1000 spectrophotometer (Peqlab, Erlangen, Germany). Reverse transcription was performed with the Maxima First Strand cDNA Synthesis Kit for RT-PCR (Thermo Fisher Scientific). For Reverse transcription of micro RNA, the MystiCq microRNA cDNA Synthesis Mix (Sigma-Aldrich) was used. RNA expression of in Table 1 listed targets was analyzed using PowerUp SYBR Green Master Mix (Applied Biosystems, Thermo Fisher Scientific) on a QuantStudio 3 PCR Detection System (Applied Biosystems, Thermo Fisher Scientific) and normalized to TBP. Primers are listed in Table 1. SNORD44 primer was purchased from Sigma.

**Table 1:** List of primers

	Forward (5'–3')	Reverse (5'–3')
TBP	GCATCACTGTTTCTTGGCGT	CGCTGGAACTCGTCTCACTA
PPTC7	CGAGGGAGTCGTCTTGAGC	GCCGTCAGGATAATGTCTCCTA
HIF-1α	GCTGGCCCCAGCCGCTGGAG	GAGTGCAGGGTCAGCACTAC
HIF-2α	AAGCCTTGGAGGGTTTCATT	TGCTGGATTGGTTCACACAT
ARNT	ACTACTGCCAACCCCGAAAT	CTCTGGACAATGGCTCCTCC
GLUT1	TCACTGTGCTCCTGGTTCTG	CCTGTGCTCCTGAGAGATCC
HK2	GTGAATCGGAGAGGTCCCAC	GCTAACTTCGGCCACAGGAT
PFKL	TCGACTGCAGGACCAATGTC	AGCTTCTCCGACAACCACAG
LDHA	ACGTCAGCAAGAGGGAGAAA	CGCTTCCAATAACACGGTTT
PFKFB1	GAAACCCAGTACACCCCCTG	TTCTGCAACCTGGTTTGGGT
PFKFB2	AGGCAGGGAGGGATCTTAGG	CGATCAGAGTCGGGGAGTTG
PFKFB3	CAGCTGCCTGGACAAAACAT	GAGGGCAGGACACAAGCTAA
PFKFB4	GGGTGCCTCTTGGCCTTAAA	GCCCACACGGCATACTTTTC
miR-193a-3p	AACTGGCCTACAAAGTCCCAGT	MystiCq Universal Primer

### Seahorse

The extracellular acidification rate (ECAR) was analyzed using a Seahorse 96 extracellular flux analyzer (Agilent, Santa Clara, USA). Human macrophages were carefully scraped of the plates after treatment and seeded on a Seahorse Cell Culture Microplate at the day of measurement and equilibrated for 1 h before measurement in XF RPMI medium supplemented with 2 mM glutamine and 1 mM pyruvate. Basal acidification was measured and glucose (Agilent) was injected to a final concentration of 10 mM. The difference between basal acidification and glucose induced acidification was considered as measure for glycolysis.

### Statistics

Statistics were performed with GraphPad Prism 8.2.1. Data are expressed as mean values ± SEM. Statistically significant differences were calculated after analysis of variance (ANOVA) and Bonferroni’s test or Students *t* test; *p* ≤ 0.05 was considered significant.

## Results

### miR-193a-3p increased Akt phosphorylation under hypoxia

**Fig. 1:**
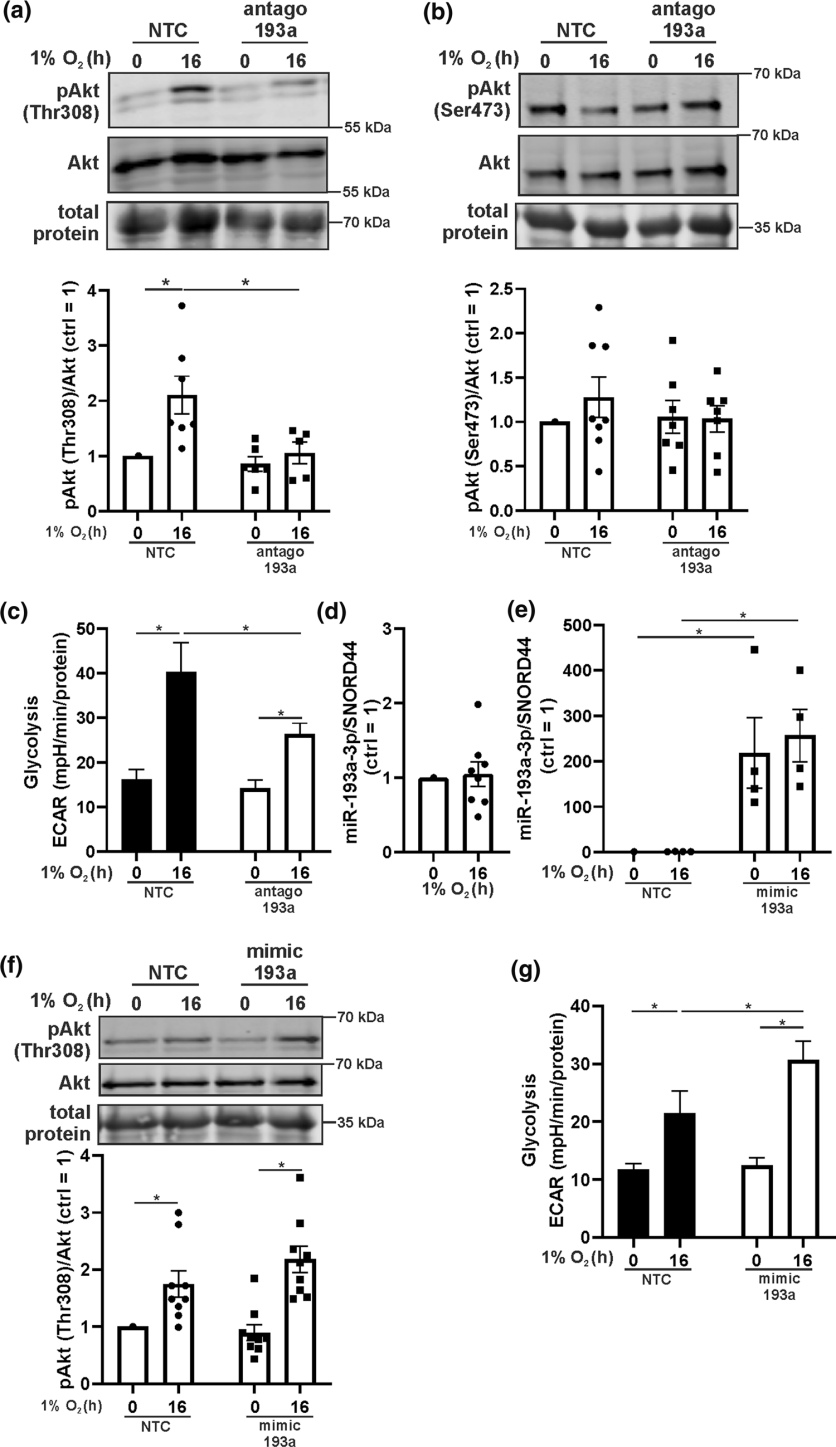
miR-193a-3p supports Akt phosphorylation and glycolysis under hypoxia. **a**, **b** Primary human macrophages were transfected with antagomir against miR-193a-3p (antago 193a) or a non-targeting control (NTC) and incubated for 16 h under hypoxia (1% O_2_). Phosphorylation of Akt at threonine 308 (Thr308) and serine 473 (Ser473) was determined by Western analysis and normalized to total Akt. **c** The extracellular acidification rate (ECAR) of normoxic and hypoxic NTC and antago 193a macrophages was measured by Seahorse. Glycolysis-dependent acidification was calculated by subtracting basal acidification from glucose-induced acidification. **d** mir-193a-3p abundance under normoxia and hypoxia (16 h, 1% O_2_) was analyzed by real time PCR and normalized to SNORD44. **e** Human macrophages were transfected with a miR-193a-3p mimic (mimic 193a) or a control (NTC) and incubated under normoxia or hypoxia. Abundance of miR-193a-3p was determined by real time PCR and data were normalized to SNORD44. **f** Akt threonine 308 phosphorylation in normoxic and hypoxic NTC and mimic 193a cells was assessed by Western analyses and normalized to total Akt. **g** Glycolysis-dependent acidification of normoxic, hypoxic NTC, and mimic 193a macrophages was measured by Seahorse. All data are expressed as mean values ± SEM, *p ≤ 0.05. Each dot represents an individual donor

Under hypoxia cells adjust their metabolism by decreasing oxidative phosphorylation and increasing glycolysis. Besides hypoxia-inducible factors (HIF), Akt is activated by oxygen deprivation and a link between Akt and miR-193a-3p has been suggested [29]. We questioned whether miR-193a-3p could regulate Akt phosphorylation under hypoxia in primary human macrophages. Monocytes were isolated from human blood and differentiated to macrophages with serum for 7 days. Afterwards cells were transfected with an antagomir for miR-193a-3p (antago 193a) or a non-targeting control (NTC), incubated for 16 h under hypoxia (1% O_2_), and analyzed for Akt phosphorylation by Western analysis (Fig. 1a, b). Hypoxia significantly increased Akt phosphorylation at threonine 308 (Thr308), while serine 473 (Ser473) remained unaltered. Cells treated with antago 193a failed to increase phosphorylation of Thr308 under hypoxia. Akt is engaged in several metabolic processes, including glycolysis. We became interested to see whether decreased Akt phosphorylation seen with antago 193a affects glycolysis (Fig. 1c, Suppl. Fig. 2a). Measuring extracellular acidification (ECAR) by Seahorse showed a significantly increased glycolysis under hypoxia in control cells, which was absent with the miR-193a-3p antagomir. These observations implied a direct connection between miR-193a-3p and induction of glycolysis under hypoxia. Real time PCR analyses showed no altered miR-193a-3p expression, comparing normoxia and hypoxia (Fig. 1d). To explore whether induction of miR-193a-3p increases Akt phosphorylation, human macrophages were transfected with a miR-193a-3p mimic (mimic 193a) or NTC and expression of the miR was validated by real time PCR (Fig. 1e). As seen before, in control cells hypoxia increased Akt phosphorylation (Fig. 1f). The mir-193a-3p mimic had no effect on Akt phosphorylation in normoxic cells but further increased Akt phosphorylation under hypoxia compared to NTC. We then determined the effect of mir-193a-3p mimic on glycolysis, by Seahorse analysis (Fig. 1g, Suppl. Fig. 2b). Glycolysis increased in hypoxic NTC cells and even more in hypoxic mir-193a-3p mimic cells, which apparently correlates with phospho-Akt. These experiments suggest that basal expression of miR-193a-3p is crucial for Akt phosphorylation as well as increased glycolysis under hypoxia and provoke the hypothesis that miR-193a-3p regulates the level of phospho-Akt by affecting a phosphatase. If correct, the postulated phosphatase should increase when miR-193a-3p is inhibited and consequently should facilitate dephosphorylation of Akt under hypoxia.

### PPTC7 is decreased by miR-193a-3p

**Fig. 2:**
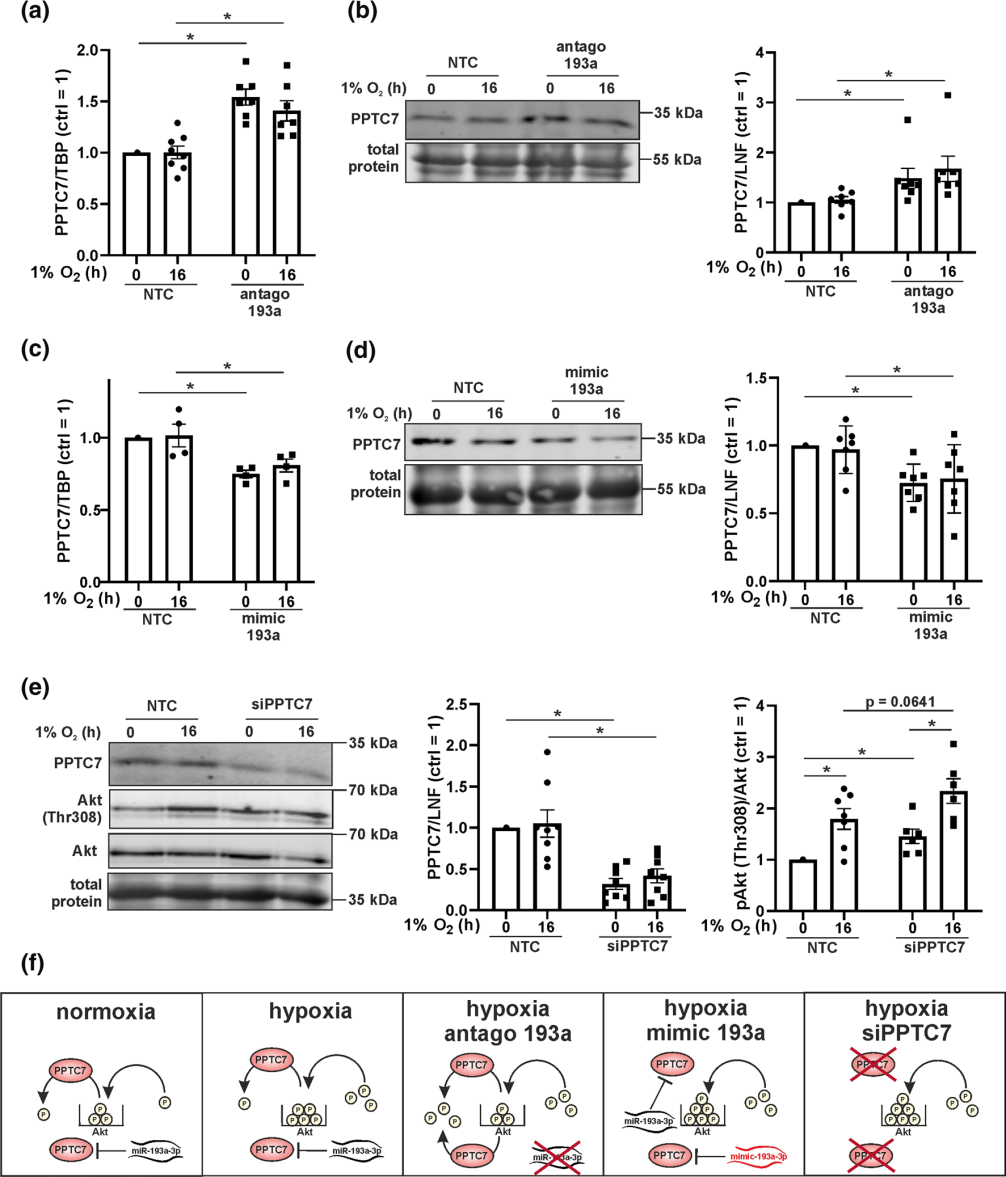
miR-193a-3p regulates PPTC7.** a** Primary human macrophages were transfected with antagomir against miR-193a-3p (antago 193a) or a non-targeting control (NTC) and incubated for 16 h under hypoxia (1% O_2_). Afterwards mRNA expression of PPTC7 was measured by real time PCR and normalized to TATA box binding protein (TBP). **b** PPTC7 protein expression under normoxia and hypoxia of NTC and antago 193a macrophages was determined by Western analysis and normalized to the lane normalization factor (LNF). **c** Human macrophages were transfected with a mimic for miR-193a-3p (mimic 193a) or a control (NTC) and incubated under normoxia or hypoxia. mRNA expression of PPTC7 was assessed by real time PCR and normalized to TATA box binding protein (TBP). **d** PPTC7 protein expression was analyzed by Western blot and normalized to LNF. **e** Macrophages were transfected with a siRNA against PPTC7 (siPPTC7) or a control (NTC) and incubated for indicated timepoints under hypoxia. PPTC7, Akt, and phosphorylation of threonine 308 (Thr308) were assessed by Western analyses. PPTC7 was normalized to LNF. Phosphorylated Akt was normalized to total Akt. **f** Scheme describing the proposed function of miR-193a-3p and PPTC7. All data are expressed as mean values ± SEM, *p ≤ 0.05. Each dot represents an individual donor

To verify our hypothesis, we analyzed mRNA expression of phosphatases, which are either known to regulate Akt or are predicted miR-193a-3p targets (Fig. 2a and Suppl. Fig. 3a–g). From 7 analyzed phosphatases only PPP2R5C and PPTC7 increased in miR-193a-3p antagomir-treated cells and only PPTC7 was elevated with the antagomir being present under hypoxia and normoxia (Fig. 2a). Moreover, PPTC7 was predicted as a miR-193a-3p target by Targetscan (http://www.targetscan.org/vert_72/, 08/2021) but so far is not known to regulate Akt (predicted target sequence shown in Suppl. Fig. 3 h). Following mRNA analysis of PPTC7 we determined protein expression by Western blotting (Fig. 2b). Corroborating mRNA data, the miR-193a-3p antagomir elevated PPTC7 protein under normoxia as well as hypoxia. To strengthen the idea that PPTC7 is a direct miR-193a-3p target, macrophages were transfected with a miR-193a-3p mimic followed by analysis of its mRNA and protein of PPTC7 abundance (Fig. 2c, d). In miR-193a-3p mimic cells PPTC7 mRNA and protein expression were significantly lower than in control transfected macrophages, while no difference was apparent comparing normoxia and hypoxia.

To proof that Akt phosphorylation demands PPTC7, we transfected macrophages with siRNA against PPTC7 and analyzed Thr308 phosphorylation of Akt. The PPTC7 siRNA-mediated knockdown was validated by Western analysis (Fig. 2e). Akt phosphorylation increased under hypoxia and the PPTC7 knockdown enhanced Akt phosphorylation at Thr308 under both, normoxia and hypoxia compared to NTC. These data support a model, where Akt phosphorylation is adjusted by miR-193a-3p and PPTC7 (Fig. 2f). Under hypoxia phosphorylation of Akt increases presumably by enhanced phosphokinase activity, despite the action of miR-193a-3p. When mir-193a-3p is inhibited, the protein amount of PPTC7 increases and counteracts enhanced hypoxic-induced Akt phosphorylation. In contrast, the miR-193a-3p mimic or the knockdown of PPTC7 facilitate phosphorylation of Akt under hypoxia. In a next step we searched for the kinase provoking Thr308 phosphorylation of Akt under hypoxia and asked whether Akt modulates glycolysis under hypoxia.

### Phospho-Akt is crucial to increase glycolysis under hypoxia

**Fig. 3:**
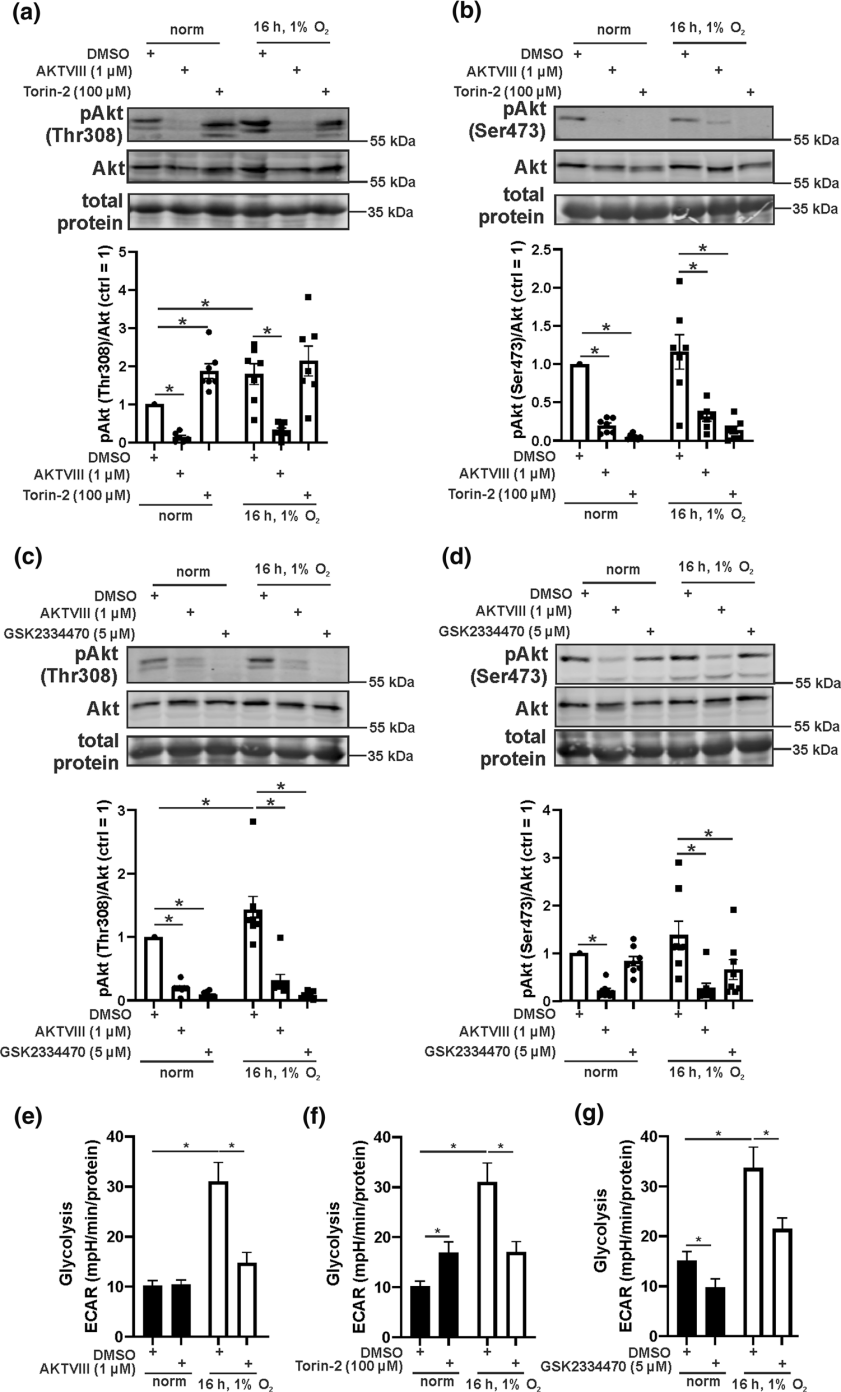
Inhibition of the Akt pathway reduces hypoxic glycolysis.** a**,** b** Primary human macrophages were treated with an inhibitor for Akt (AKTVIII) or mTOR (Torin-2) 1 h prior to hypoxic incubation (16 h, 1% O_2_). Akt phosphorylation at threonine 308 (Thr308) and serine 473 (Ser473) was analyzed by Western analysis and normalized to total Akt. **c **and** d** Macrophages were treated with an inhibitor for Akt (AKTVIII) or PDPK1 (GSK2334470) 1 h prior hypoxic incubation. Thr308 and Ser473 phosphorylation of Akt was followed by Western analysis and normalized to total Akt. **e**–**g** Primary human macrophages were cultured as described above and extracellular acidification (ECAR) was assessed by Seahorse. All data are expressed as mean values ± SEM, *p ≤ 0.05. Each dot represents an individual donor

In search for the kinase we used various inhibitors and analyzed phospho-Akt by Western analysis. First, mTOR and Akt were inhibited by Torin-2 and AKTVIII, while DMSO served as a control (Fig. 3a, b). DMSO allowed hypoxia to phosphorylate of Akt at Thr308 but not Ser473. AKTVIII prevented phosphorylation at either site, both under normoxia and hypoxia. Torin-2 increased Thr308 phosphorylation under normoxia but had no effect under hypoxia, while Ser473 phosphorylation was completely abolished under normoxia and hypoxia. Torin-2 was proposed to be more active towards mTORC1 but reducing Ser473 phosphorylation indicates a major effect of mTORC2. PDPK1 is known to phosphorylate Akt at Thr308 and was antagonized by GSK2334470 (Fig. 3c). Inhibition of PDPK1 reduced phosphorylation at Thr308 under normoxia and hypoxia but had no effect on Ser473 under normoxia, while decreased phosphorylation was observed under hypoxia. To link potential kinases via Akt to glycolysis, Seahorse experiments with inhibitors were performed. Hypoxia increased the glycolytic flux (Fig. 3e–g) and inhibition of Akt by AKTVIII significantly attenuated hypoxic-driven glycolysis but showed no inhibitory potency under normoxia (Fig. 3e). Interestingly, glycolysis increased upon Torin-2 treatment under normoxia, correlating with higher rates of Thr308 phosphorylation (Fig. 3f). Under hypoxia, Torin-2 significantly reduced glycolysis. Blocking PDPK1 with GSK2334470 decreased glycolysis under normoxia and hypoxia, which correlates with reduced Akt-Thr308 phosphorylation (Fig. 3g). These results let us conclude that PDPK1 phosphorylates Akt at Thr308, which increases glycolysis under hypoxia. Glycolysis might be increased via mTORC1, which previously was described as a regulator of the glycolytic flux [31]. However, Akt activation via mTORC2 has also to be considered. Since HIF-1α is a major regulator of glycolysis under hypoxia, we went on to estimate the effect of miR-193a-3p and Akt on HIF-1α abundance and function to explore the role of HIF on glycolysis and Akt activation.

### Akt regulates glycolysis HIF-independently

**Fig. 4:**
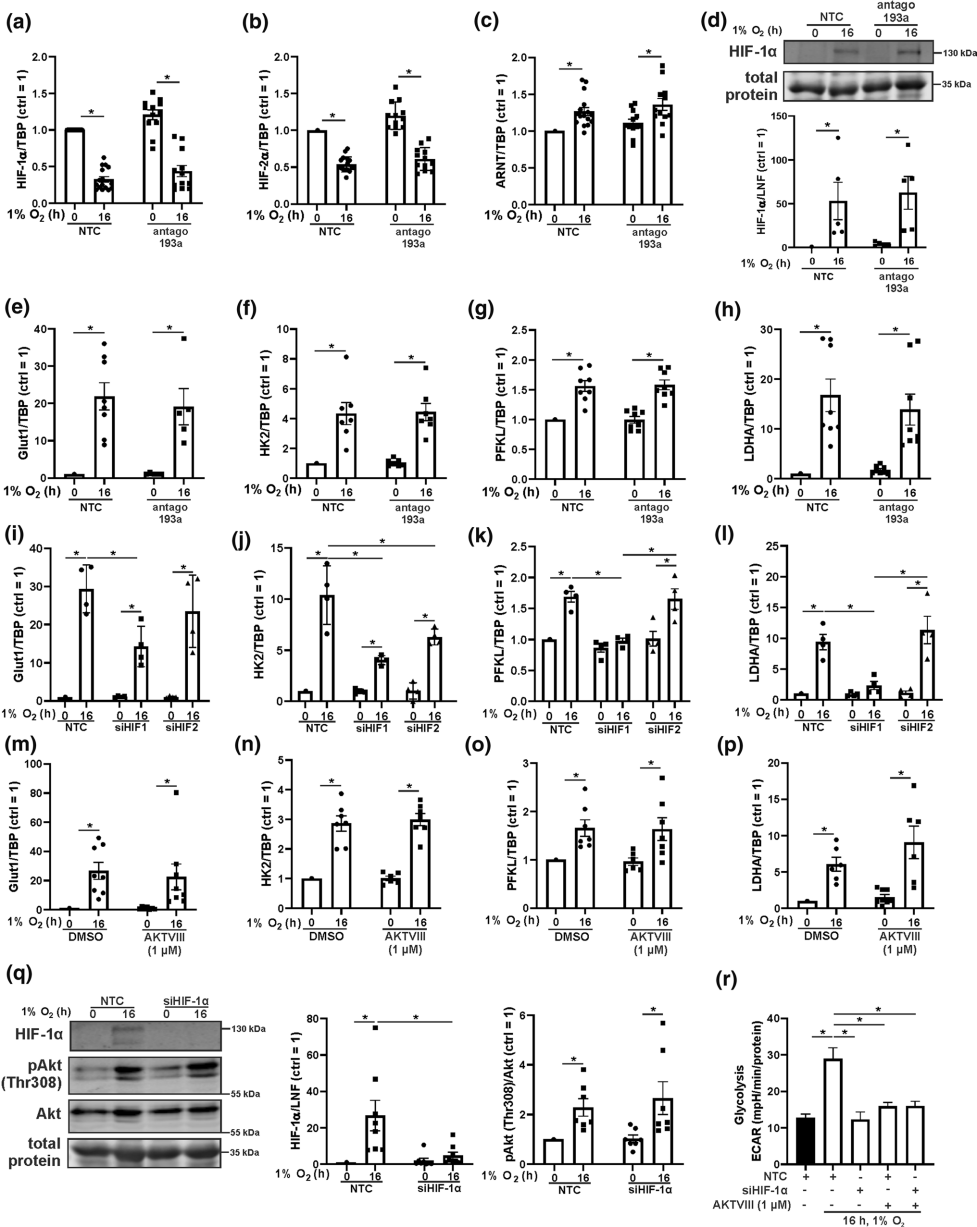
Akt and miR-193a-3p do not regulate HIF.** a**–**c** Primary human macrophages were transfected with antagomir against miR-193a-3p (antago 193a) or a non-targeting control (NTC) and incubated for 16 h under hypoxia (1% O_2_). Afterwards mRNA expression of HIF-1α, HIF-2α, and ARNT was measured by real time PCR and normalized to TATA box binding protein (TBP). **d** Macrophages were treated as described above, HIF-1α was assessed by Western analyses and normalized to the lane normalization factor (LNF). **e**–**h** miR-193a-3p was inhibited in human macrophages and hypoxic induction of Glut1, HK2, PFKL, as well as LDHA mRNA was measured, and normalized to TBP. **i**–**l** Macrophages were transfected with siRNA against HIF-1α (siHIF-1α), HIF-2α (siHIF-2α), or a control (NTC) and incubated for times indicated under hypoxia. mRNA of Glut1, HK2, PFKL, and LDHA was measured and normalized to TBP. **m**–**p** Cells were treated with AKTVIII and hypoxic mRNA induction of Glut1, HK2, PFKL, and LDHA was measured, and normalized to TBP. **q** HIF-1α was knocked down by siRNA and cells were incubated for 16 h under hypoxia. HIF-1α, phosphorylated Akt (Thr308), and total Akt was determined by Western analysis. HIF-1α was normalized to LNF. Phosphorylated Akt was normalized to total Akt. **r** Macrophages were transfected with siRNA against HIF-1α (siHIF-1α) or a control (NTC), treated with AKTVIII and incubated for 16 h under hypoxia. Extracellular acidification (ECAR) was assessed by Seahorse. All data are expressed as mean values ± SEM, *p ≤ 0.05. Each dot represents an individual donor

To understand potential effects of miR-193a-3p on HIF-expression, the miR was inhibited followed by analysis of mRNA levels of HIF-1α, HIF-2α, and ARNT (Fig. 4a–c). HIF-1α and HIF-2α mRNAs significantly decreased under hypoxia, while ARNT was slightly increased. Importantly, antagonizing miR-193a-3p was without effect. In analogy, HIF-1α stabilization was not affected by miR-193a-3p (Fig. 4d). Further experiments analyzed the impact of miR-193a-3p on the expression of classical HIF-1 target genes, e.g., Glut1, HK2, PFKL, and LDHA (Fig. 4e–h). These genes were responsive to hypoxia and miR-193a-3p did not interfere with their induction. To validate Glut1, HK2, PFKL, and LDHA as HIF-1 targets, we knocked down HIF-1α (siHIF-1α) and HIF-2α (siHIF-2α) in macrophages (Fig. 4i–l). Except HK2, all genes increased under hypoxia in control and HIF-2α knockdown cells but the expression was significantly reduced when HIF-1α was knocked down. While these results verified Glut1, PFKL, and LDHA as HIF-1 targets in human macrophages, HK2 decreased in HIF-1α and HIF-2α knockdown macrophages and thus, can be considered a target of both isoforms. To see whether Akt affects HIF-1α, we followed HIF target gene expression in AKTVIII treated cells (Fig. 4m–p). Hypoxic induction of Glut1, HK2, PFKL, and LDHA remained unaffected when Akt was blocked. We then elucidated the role of HIF-1α in Akt activation (Fig. 4q). HIF-1α was knocked down by siRNA and macrophages were incubated under hypoxia for 16 h. An efficient knockdown of HIF-1α was validated by Western analyses. Akt phosphorylation at Thr308 increased in hypoxic control and siHIF-1α cells to the same extend. These data suggest that HIF-1α and miR193a-3p/Akt are involved in hypoxic regulation of glycolysis but act via different pathways. To determine the impact of HIF-1α and Akt on hypoxia-induced glycolysis we performed Seahorse experiments in control, HIF-1α knockdown, and AKTVIII treated cells (Fig. 4r). Hypoxia-driven glycolysis was significantly reduced by siHIF-1α and Akt inhibition. Both treatments lowered glycolysis to basal values, indicating an equal importance of HIF-1α and Akt in enhancing glycolysis under hypoxia. Conclusively, Akt and indirectly miR-193a-3p do not regulate glycolysis under hypoxia via enhanced HIF-signaling, although both appeared of equal importance. Another important regulator of glycolysis is 6-phosphofructo-2-kinase/fructose-2,6-bisphosphatases (PFKFB), which converts fructose 6-phosphate to fructose 2,6-bisphosphate and vice versa. Fructose 2,6-bisphosphate in turn increases the activity of phosphofructo-kinase (PFK), which catalyzes the rate limiting glycolytic step from fructose 6-phosphate to fructose 1,6-bisphosphate.

### PFKFB expression under hypoxia

**Fig. 5:**
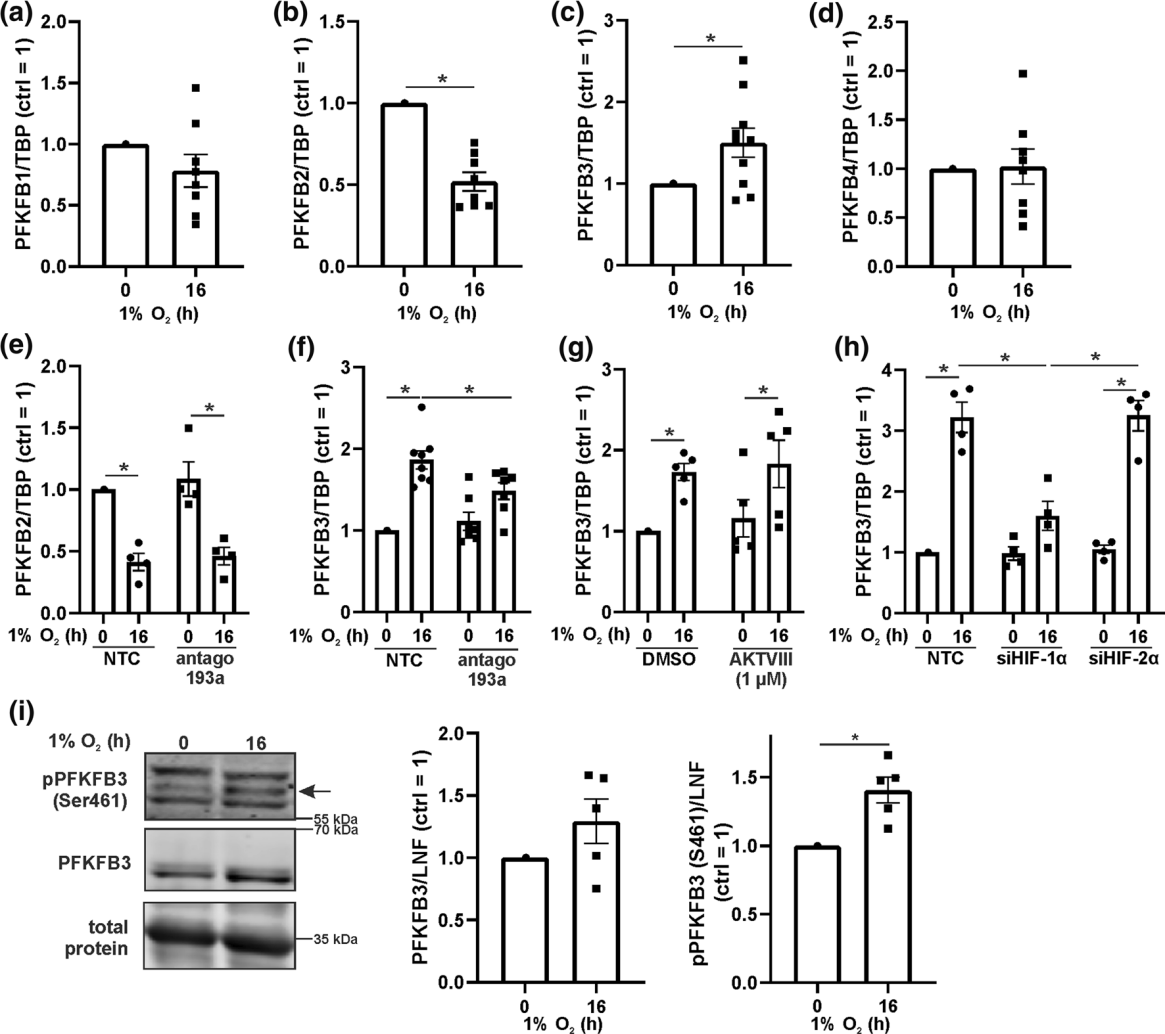
Phosphorylated PFKFB3 increases under hypoxia.** a**–**d** Primary human macrophages were incubated for 16 h under hypoxia and PFKFB1-4 were measured at mRNA level. Data were normalized to TATA box binding protein (TBP). **e**–**f** Cells were transfected with antagomir against miR-193a-3p (antago 193a) or a non-targeting control (NTC) and incubated for 16 h under hypoxia (1% O_2_). mRNA of PFKFB2 und PFKFB3 was assessed by real time PCR and normalized to TATA box binding protein (TBP). **g** Akt was inhibited by AKTVIII and PFKFB3 mRNA was measured by real time PCR and normalized to TBP. **h** Human macrophages were transfected with siRNA against HIF-1α (siHIF-1α), HIF-2α (siHIF-2α), or a control (NTC) and incubated for times indicated under hypoxia. mRNA of PFKFB3 was measured and normalized to TBP. **i**–**j** Human macrophages were incubated for 16 h under hypoxia and total as well as phosphorylated PFKFB2 and PFKFB3 were assessed by Western analysis. All data are expressed as mean values ± SEM, *p ≤ 0.05. Each dot represents an individual donor

Fist we assessed mRNA expression of PFKFB isoenzymes 1–4 (Fig. 5a–d). PFKFB1 and 4 were not regulated under hypoxia. Levels of PFKFB2 decreased, while PFKFB3 significantly increased after 16 h of hypoxia. We then analyzed the impact of miR-193a-3p on PFKFB2 and 3 expressions, using the miR-193a-3p antagomir (Fig. 5e and f). RNA expression of PFKFB2 decreased under hypoxia, irrespective of miR-193a-3p. In contrast, hypoxia elevated PFKFB3 levels in control and antagomir-treated cells with the notion that the mRNA increase was lower with miR-193a-3p being antagonized. Whether this accounts for any physiological relevance remains questionable. We also analyzed PFKFB3 mRNA expression by blocking Akt with AKTVIII (Fig. 5g). As the Akt inhibitor was without effect, this pathway apparently does not impinge on PFKFB3 expression. To clarify the role of HIF, we measured PFKFB3 in controls and corresponding knockdown samples (Fig. 5h). Hypoxia-induced PFKFB3 expression was absent in siHIF-1α cells but not in siHIF-2α cells, which suggest PFKB3 as an HIF-1 target. This was followed by Western analysis of PFKFB3 expression and its degree of phosphorylation (Fig. 5i). PFKFB3 protein amount slightly but not significantly increased without reaching significance and thus, not fully recapitulated mRNA expression data. In contrast, phosphorylation of PFKFB3 at serine 461 (Ser461) significantly increased under hypoxia, which points to enhanced enzyme activity. As PFKFB3 was previously described as an Akt target, it seems logic that enhanced PFKFB3 phosphorylation could facilitate the miR-193a-3p/Akt response to enhance glycolysis under hypoxia.

### PFKFB3 is regulated by the miR-193a-3p/Akt/mTOR axis under hypoxia

**Fig. 6:**
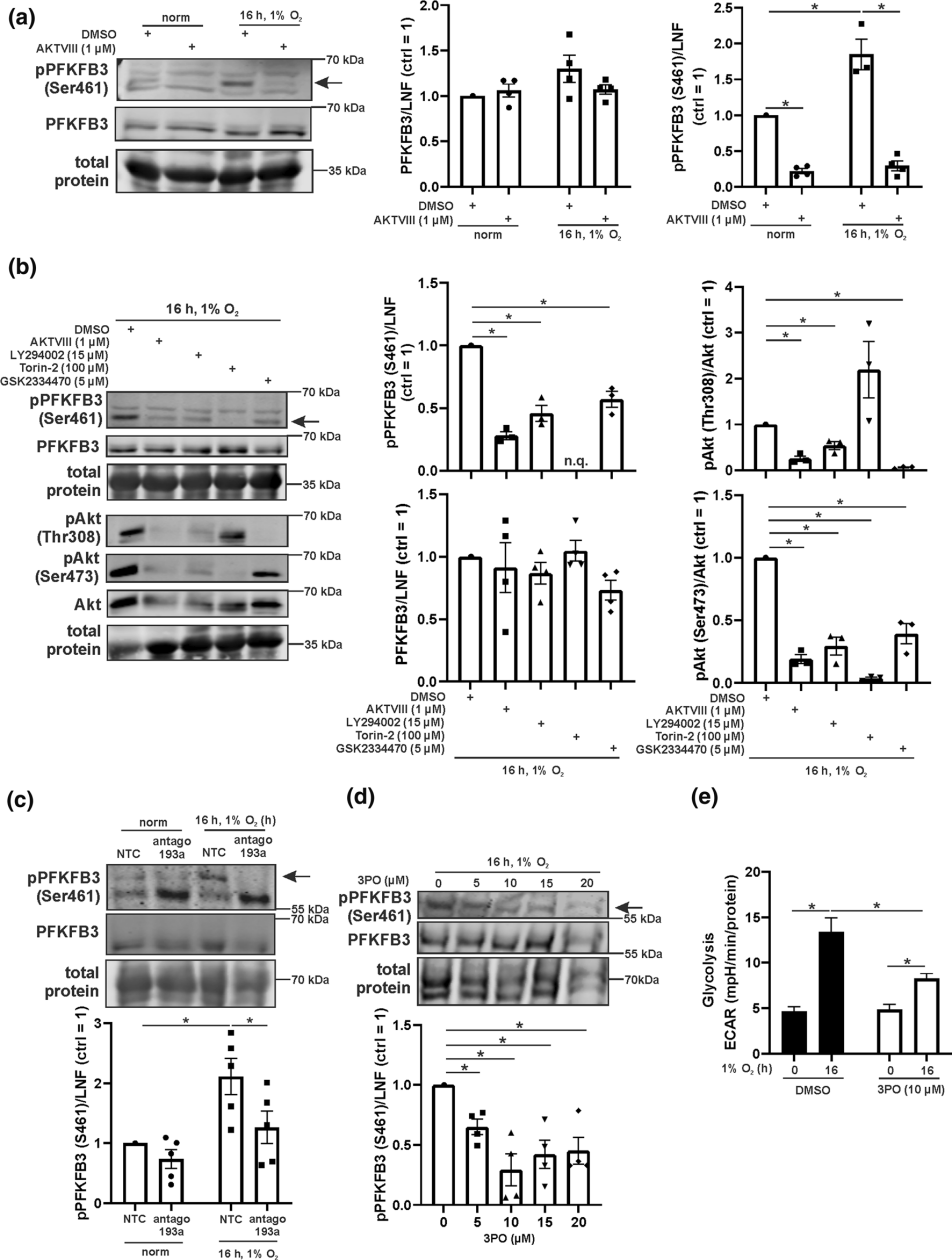
The PI3K/Akt/mTOR cascade regulates PFKFB3 phosphorylation.** a** Primary human macrophages were treated with an Akt inhibitor (AktVIII) 1 h prior to hypoxic incubation (16 h, 1% O_2_). Phosphorylated (serine 461, Ser461) and total PFKFB3 was determined by Western analysis and normalized to lane normalization factor (LNF). **b** macrophages were treated inhibitors for Akt (AKTVIII), PI3K (LY294002), mTOR (Torin-2), and PDPK1 (GSK2334470) 1 h prior to hypoxic incubation. Phosphorylated (Ser461) and total PFKFB3 was determined by Western analysis and normalized to LNF. Phosphorylated Akt (Thr308 and Ser473) was assessed and normalized to total Akt. **c** Macrophages were transfected with antagomir against miR-193a-3p (antago 193a) or a non-targeting control (NTC) and incubated for 16 h under hypoxia (1% O_2_). Phosphorylated (Ser461) and total PFKFB3 was determined by Western analysis and normalized to LNF. **d** Cells were treated with increasing concentrations of the PFKFB3 inhibitor 3PO 1 h prior to hypoxic incubation. Phosphorylated (Ser461) and total PFKFB3 was measured by Western analysis and normalized to LNF. **e** Macrophages were treated with 3PO 1 h prior to hypoxic incubation. Extracellular acidification rate (ECAR) was assessed by Seahorse. All data are expressed as mean values ± SEM, *p ≤ 0.05. Each dot represents an individual donor

To analyze whether Akt affects PFKFB3 phosphorylation we used AKTVIII in human macrophages and incubated them for 16 h under hypoxia (Fig. 6a). While total PFKFB3 expression remained unaltered, phospho-PFKFB3 significantly increased under hypoxia and this increase was fully blocked by AKTVIII. For a more detailed analysis we interfered with different enzymes of the Akt signaling cascade (Fig. 6b). Inhibition of Akt (AKTVIII), PI3K (LY294002), mTOR (Torin-2), and PDPK1 (GSK2334470) reduced PFKFB3 phosphorylation under hypoxia. In addition, phosphorylation of Akt was monitored. Akt phosphorylation at Thr308 was significantly blocked by all inhibitors except Torin-2, while Ser473 phosphorylation was prevented by all antagonists. Because Torin-2 completely vanished PFKFB3 phosphorylation no quantification was possible. This effect can be explained by efficient inhibition of mTORC2 and consequently Akt Ser473 phosphorylation, which is a perquisite for Akt function. Alternatively, PFKFB3 was identified as mTORC1 target and consequently, inhibition by Torin-2 may account for reduced PFKFB3 phosphorylation [37]. It appears that PI3K/Akt/mTOR signaling is crucial for PFKFB3 activation under hypoxia. As proof of principle, we explored whether miR-193a-3p inhibition reduces PFKFB3 phosphorylation. We analyzed PFKFB3 in miR-193a-3p antagomir treated cells by Western analysis (Fig. 6c, quantification of total PFKFB3 in Suppl. Fig. 4a). Indeed, phospho-PFKFB3 significantly increased in controls under hypoxia but not when miR-193a-3p was antagonized.

### PFKFB3 increases glycolysis under hypoxia

Finally, we determined the role of PFKFB3 for glycolysis under hypoxia. Human macrophages were exposed to increasing concentrations of the PFKFB3 inhibitor 3-(3-pyridinyl)-1-(4-pyridinyl)-2-propen-1-one (3PO) and incubated under hypoxia (Fig. 6d, quantification of total PFKFB3 in Suppl. Fig. 4b). 3PO at 5 µM significantly decreased the amount of pPFKFB3. This became even more pronounced at 10 µM 3PO and higher, without altering total PFKFB3 expression. Cells were then treated with 10 µM 3PO to follow the rate of glycolysis by Seahorse measurements (Fig. 6e). Hypoxic induction of glycolysis was efficiently reduced by 3PO, without affecting the basal rate of glucose conversion in glycolysis.

**Fig. 7:**
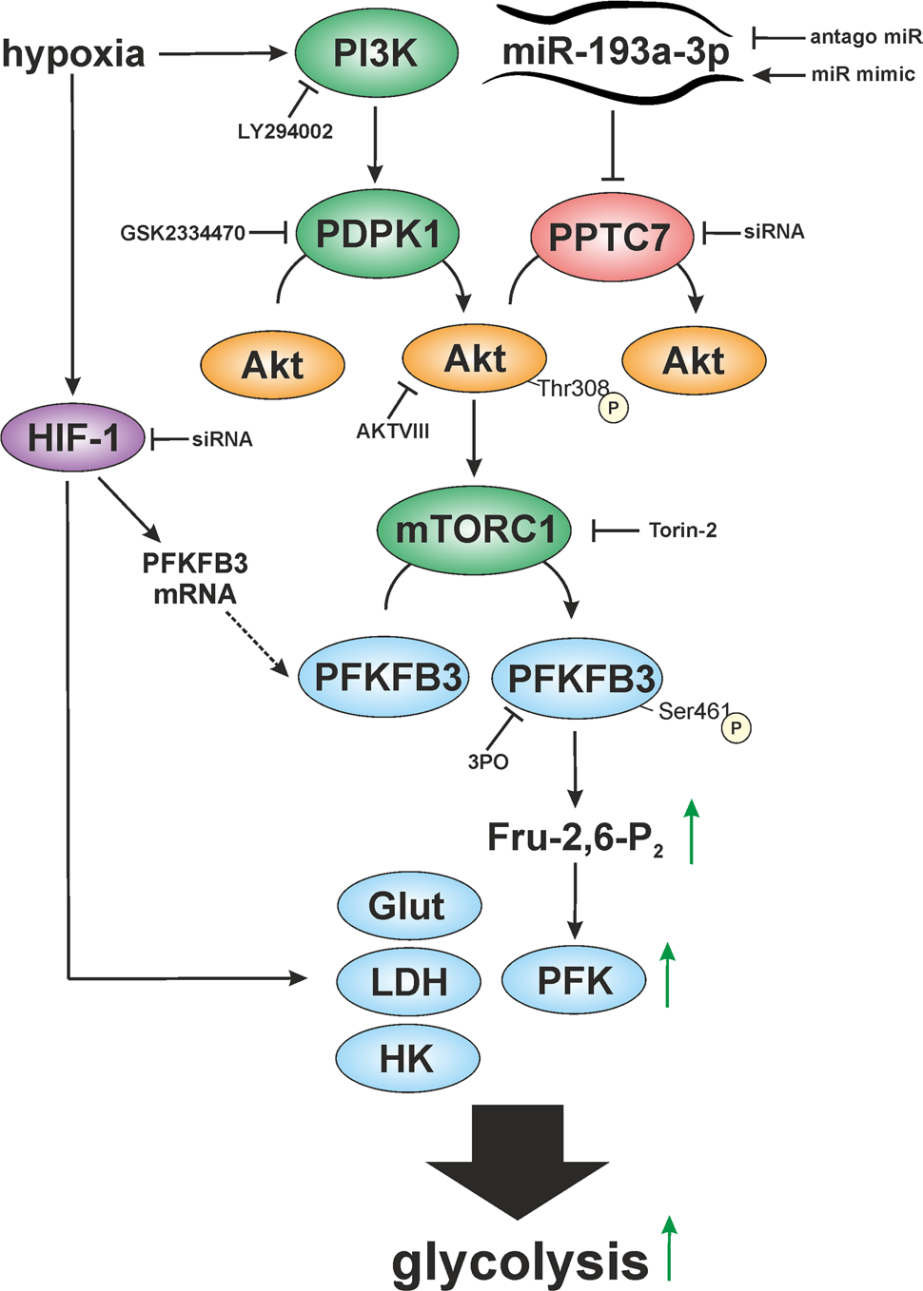
Proposed model how miR-193a-3q affects glycolysis. miR-193a-3p blocks PPTC7 expression, which allows increased Akt phosphorylation under hypoxia by PDPK1. Akt causes mTORC1 activation and subsequent PFKFB3 phosphorylation increases fructose-2,6-bisphosphate to stimulate PFK activity. This is accompanied by an HIF-1-dependent increase of glycolytic genes/proteins. In combination this enhances glycolysis under hypoxia. Interference with distinct inhibitors is indicated

In essence, miR-193a-3p affects Akt phosphorylation in human macrophages. Inhibition of miR-193a-3p counteracts hypoxia-induced Akt phosphorylation at Thr308 by increasing PPTC7. This prevents Akt-mediated PFKFB3 activation and consequently, a higher glycolytic flux rate (Fig. 7).

## Discussion

Our study provides novel information towards the functional role of miR-193a-3p in affecting the degree of Akt phosphorylation and thus, its activity. Mechanistically miR-193a-3p enhances Akt activity in primary human macrophages by decreasing the expression of the phosphatase PPTC7. Qian and coworkers showed that the long non-coding RNA PTENP1 functions as a sponge for miR-193a-3p. In their case, the lower miR-193a-3p abundance increased PTEN expression, which consequently reduced Akt phosphorylation [30]. Their observation and our study suggest that miR-193a-3p functions as a regulator of Akt activation. Receptor tyrosine–protein kinase erbB-4 (ERBB4) and ribosomal protein S6 kinase beta-2 are additional targets of miR-193a-3p [24]. ERBB4 apparently is involved in phosphorylation of PI3K and Akt and promotes proliferation of gastric cancer cells [32]. Decreasing ERBB4 expression by miR-193a-3p reduced Akt activity, which demanded the long intergenic noncoding RNA 152 [33]. Down regulation of the Akt pathway by overexpressing miR-193a was also observed by Polini and coworkers in melanoma cell lines, although the mechanism remained unexplored [34]. Overall, miR-193a-3p appears as a regulator of the Akt pathway with the ability to increase Akt activity by targeting phosphatases or decreasing Akt activity by targeting phosphokinases. There are also reports on activation of the Akt pathway by inhibition of miR-193a-3p and miR-224 in RCC by targeting alpha-2,3-sialyltransferase IV [35], effects which are difficult to reconcile with observations in macrophages.

Under hypoxia an increase in glycolysis compensates for decreased oxidative phosphorylation. The hypoxic driven increase in glycolysis in human macrophages appears as a result of miR-193a-3p in suppressing PPTC7, which adds to Akt phosphorylation and activation. In turn, Akt was crucial for phosphorylation and activation of PFKFB3, which enhances the catalytic activity of PFK by increasing the cellular fructose 2,6-bisphosphate level. Interfering with Akt signaling by, e.g., blocking PI3K, PDPK1, mTOR, or Akt reduced PFKFB3 phosphorylation under hypoxia. Inhibition of mTOR by Torin-2 completely suppressed PFKFB3 phosphorylation and prevented hypoxic induction of glycolysis, suggesting that either mTORC2-mediated Akt activation or mTORC1-mediated PFKFB3 phosphorylation accounts for induced glycolysis. Based on literature, mTORC1 is crucial for increasing glycolysis [36]. Wang et al. performed a knockdown of Raptor and Rictor in mouse embryotic fibroblasts and only the knockdown of Raptor, a component of mTORC1, suppressed phosphorylation of PFKFB3 [37]. Thus, regulation of PFKFB3 by mTORC1 appears likely. Furthermore, our data suggest that HIF- and Akt-pathways are of equal importance for an increased hypoxic glycolysis. An impact of HIF on PFKFB3 protein expression under acute hypoxia was ruled out, despite some mRNA increase. It cannot be ruled out that PFKFB3 protein is induced at later stages of hypoxia, since it was reported that metformin suppressed PFKFB3 expression by preventing HIF-1α accumulation in hepatoma cells, thereby inhibiting glycolysis and proliferation [38]. Our data indicate that hypoxia increases phosphorylation and thus activity of PFKFB3, which is crucial to increase glycolysis by producing fructose 2,6-bisphosphate. This concept was proven using 3PO to interfere with PFKFB3 phosphorylation and reducing glycolysis under hypoxia while leaving PFKFB3 expression unaltered.

As a result of our study the miR-193a-3p/PPTC7/Akt pathway is now linked to glycolysis. Previous evidence suggested PPTC7 as regulator of mitochondrial metabolism. Specifically, PPTC7 seems crucial for coenzyme Q10 (CoQ10) biosynthesis by dephosphorylating COQ7 [39]. CoQ10 prevents the accumulation of reactive oxygen species and ensures a proper function of the respiratory chain. In line, oxygen consumption increased in macrophages when miR-193a-3p was inhibited and PPTC7 increased (data not shown). Thus, PPTC7 and consequently miR-193a-3p could act as switch between oxidative phosphorylation, which is increased by PPTC7, and glycolysis, which is decreased by PPTC7 via dephosphorylation of Akt. PPTC7 knockout mice developed hypoketonic hypoglycemia in combination with elevated acylcarnitines and serum lactate [40] and shortly died after birth. These symptoms point to a disturbed mitochondrial fatty acid metabolism but could also indicate an increase in glycolysis, which may result from increased Akt and PFKFB3 phosphorylation.

In this study we described a so far unknown mechanism of hypoxic Akt phosphorylation, providing evidence that miR-193a-3p targets/downregulates the phosphatase PPTC7, which dephosphorylates Akt (Fig. 7). Lowering PPTC7 expression accounts for increased glycolysis under hypoxia by enhancing Akt activation and subsequent PFKFB3 phosphorylation. Modulating glycolysis by a miR may turn out as an interesting strategy to interfere with macrophage polarization and activation, since glycolysis increases upon stimulation with lipopolysaccharide. Besides macrophages also tumor cells increase glycolysis, a process known as the Warburg effect, and regulating miR-193a-3p may prevent changes in metabolism and thus progression towards a malignant tumor. Further studies are needed to increase our knowledge how miR-193a-3p affects metabolism and to explore how this miR is regulated.

## Supplementary Information

Below is the link to the electronic supplementary material.

Supplementary file1 (DOCX 576 KB)

## Data Availability

Not applicable.
