# Supplementary material for: miR-193a-3p increases glycolysis under hypoxia by facilitating Akt phosphorylation and PFKFB3 activation in human macrophages
Source: Cell Mol Life Sci. 2022 Jan 24;79(2):89. doi: 10.1007/s00018-022-04146-z (PMC8786749; doi:10.1007/s00018-022-04146-z)
Supplement: Supplementary file 1 — Supplementary file1 (DOCX 576 KB) [file 18_2022_4146_MOESM1_ESM.docx]

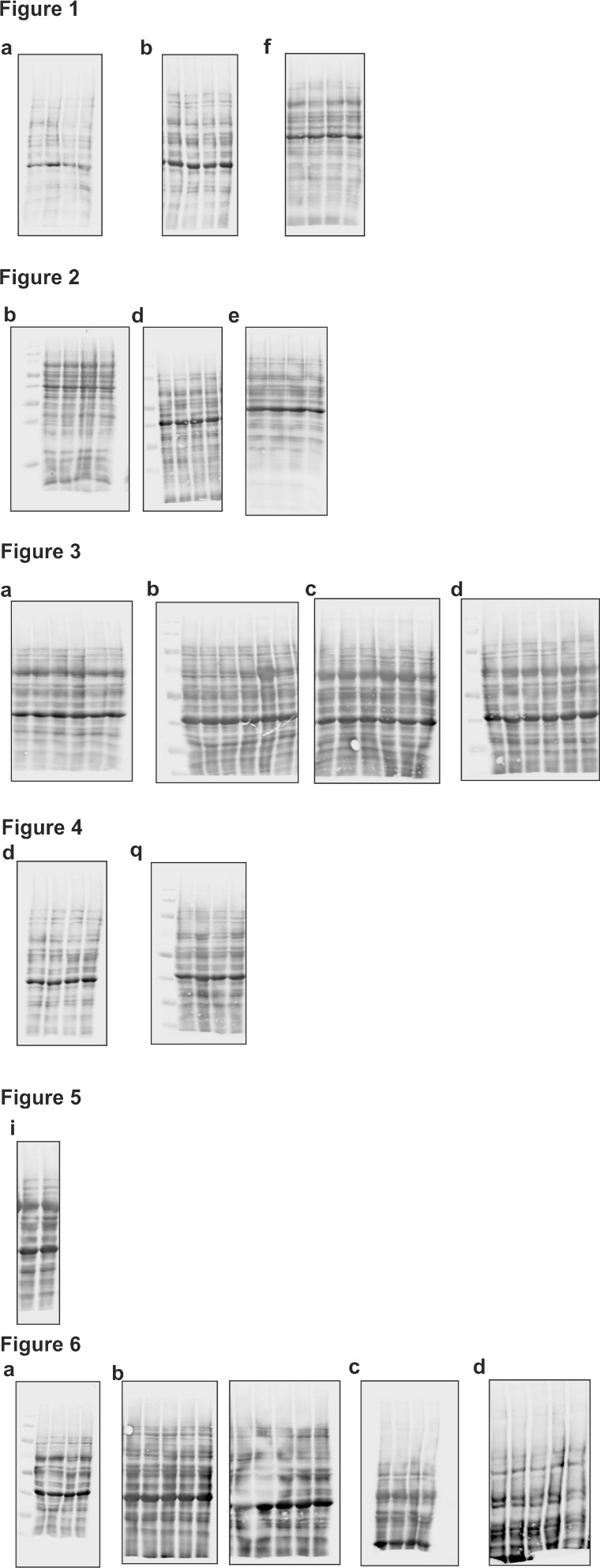


**Figure S1: Total protein stain**

Membranes were stained with Revert 700 Total Protein Stain kit before blocking. The figure shows the membrane of every blot, which was used to create the figures within this publication.

**
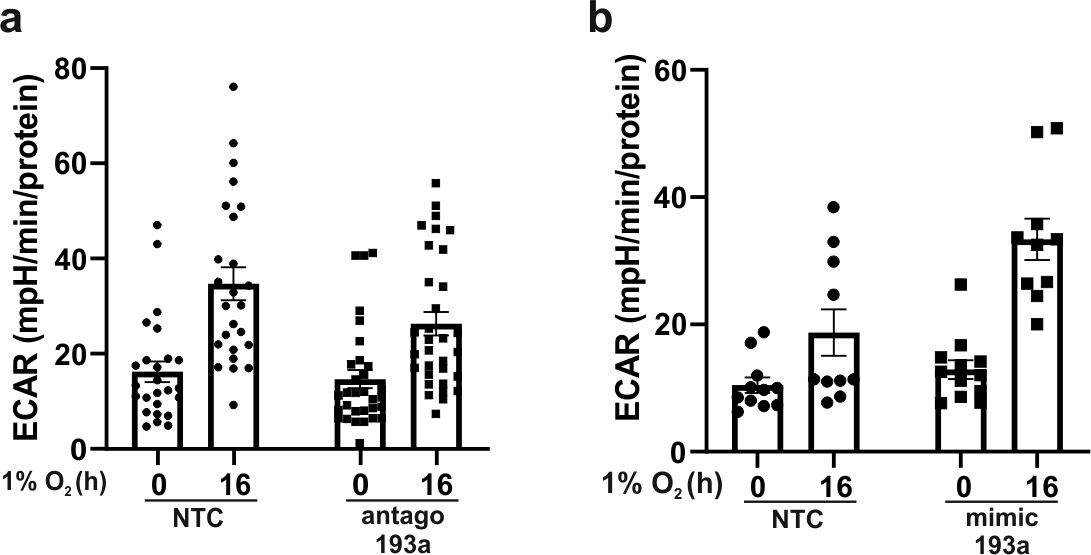
**

**Figure S2: Individual values of Fig 1c and 1g**

**a** The extracellular acidification rate (ECAR) of normoxic and hypoxic NTC and antago 193a macrophages was measured by Seahorse. Glycolysis-dependent acidification was calculated by subtracting basal acidification from glucose-induced acidification. Each dot represents one well in the measurement. 8 Buffys were used in the experiments. **b** Glycolysis-dependent acidification of normoxic, hypoxic NTC, and mimic 193a macrophages was measured by Seahorse. Each dot represents one well in the measurement. 5 Buffys were used in the experiments. All data are expressed as mean values ± SEM, *p ≤ 0.05


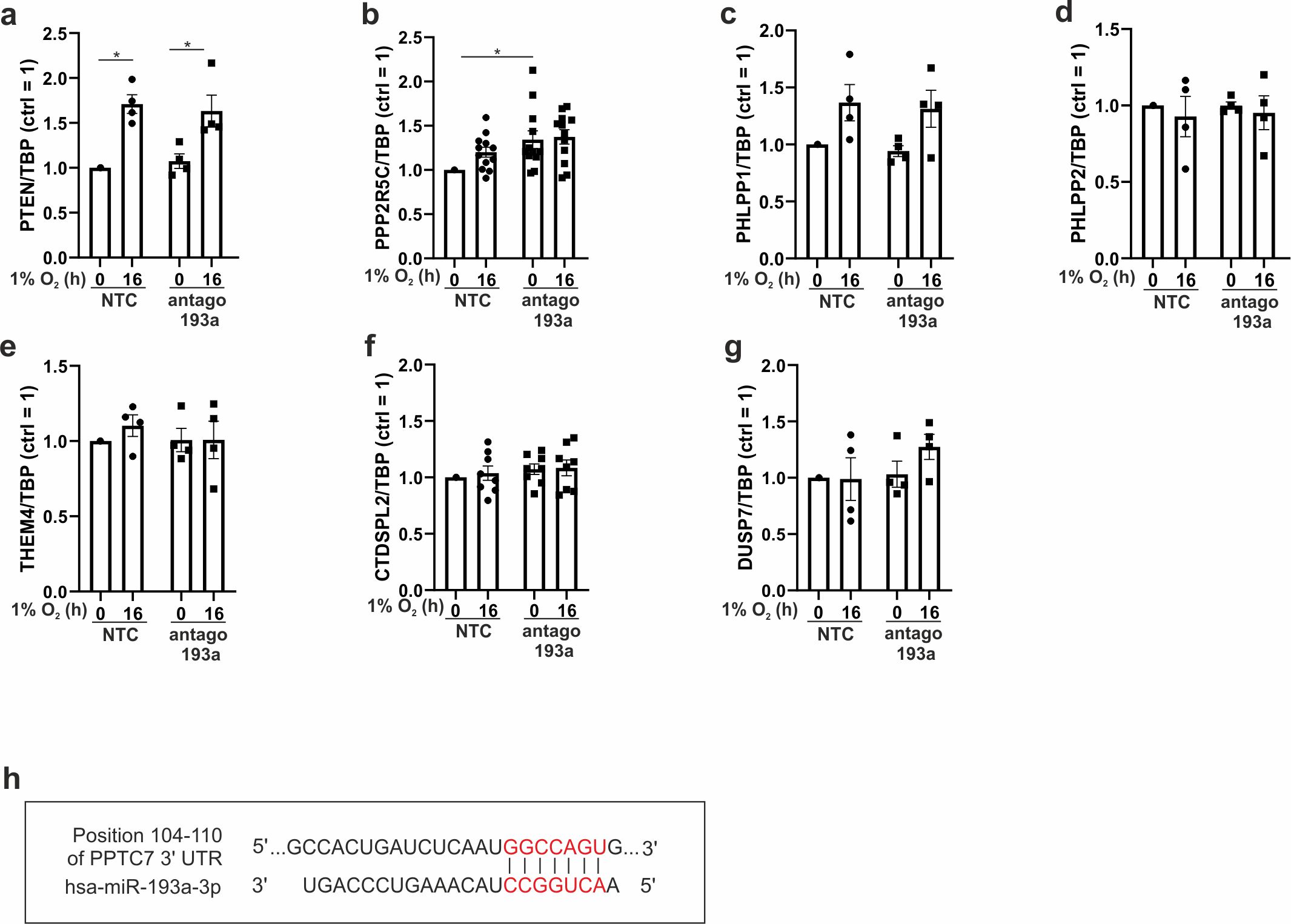


**Figure S3: mRNA regulation of Akt-regulating phosphatases**

**a-g** Primary human macrophages were transfected with antagomir against miR-193a-3p (antago 193a) or a non-targeting control (NTC) and incubated for 16 h under hypoxia (1% O_2_). mRNA of phosphatidylinositol 3,4,5-trisphosphate 3-phosphatase and dual-specificity protein phosphatase (PTEN), Serine/threonine-protein phosphatase 2A 56 kDa regulatory subunit gamma isoform (PPP2R5C), PH domain leucine-rich repeat-containing protein phosphatase (PHLPP) 1, PHLPP 2, Acyl-coenzyme A thioesterase (THEM4), CTD small phosphatase-like protein 2 (CTDSPL2), and dual specificity protein phosphatase 7 (DUSP7) was analyzed and normalized to TATA-Box binding protein (TBP). **h** Position 104-110 of the 3’ UTR sequence of protein phosphatase PTC7 homolog (PPTC7) including the predicted binding site of miR-193a-3p (red). All data are expressed as mean values ± SEM, *p ≤ 0.05


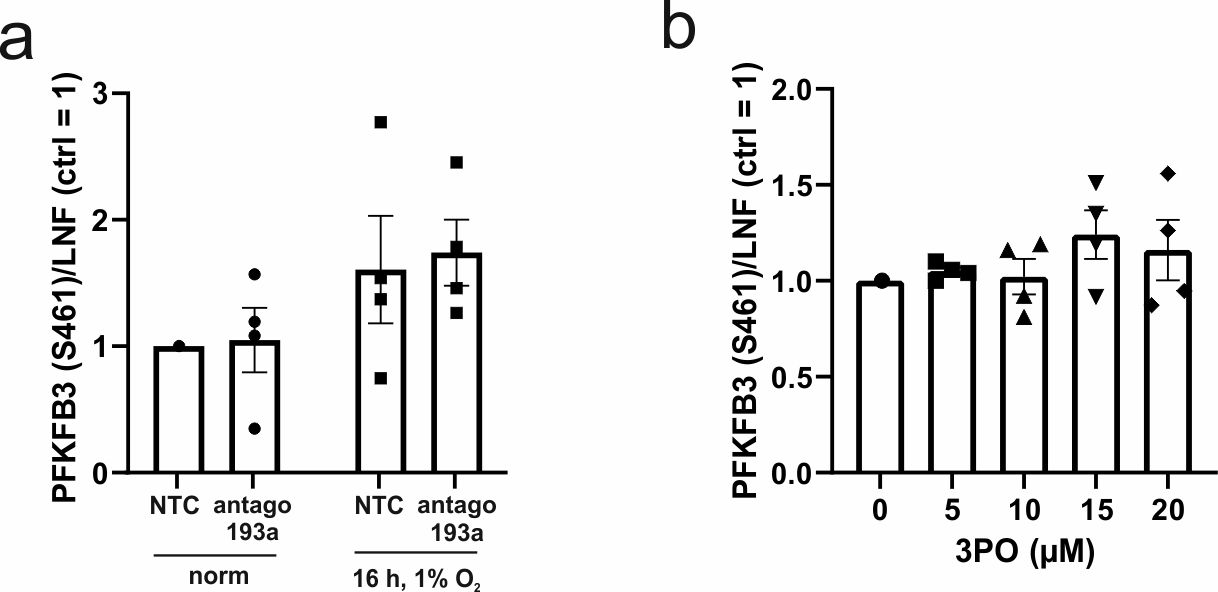


**Figure S4: Quantification total PFKFB3**

**a** Macrophages were transfected with antagomir against miR-193a-3p (antago 193a) or a non-targeting control (NTC) and incubated for 16 h under hypoxia (1% O_2_). Total PFKFB3 was determined by Western analysis and normalized to LNF. **b** Cells were treated with increasing concentrations of the PFKFB3 inhibitor 3PO 1 h prior to hypoxic incubation. Total PFKFB3 was measured by Western analysis and normalized to LNF. All data are expressed as mean values ± SEM, *p ≤ 0.05
